# Long‐Term Nutritional Benefits of Laparoscopic Pancreatoduodenectomy Over Open Surgery

**DOI:** 10.1002/ags3.70038

**Published:** 2025-05-26

**Authors:** Koki Kurahashi, Takayuki Anazawa, Kei Yamane, Kazuyuki Nagai, Satoshi Ishida, Satoshi Ogiso, Yoichiro Uchida, Takashi Ito, Takamichi Ishii, Etsuro Hatano

**Affiliations:** ^1^ Division of Hepato‐Biliary‐Pancreatic Surgery and Transplantation, Department of Surgery, Graduate School of Medicine Kyoto University Kyoto Japan

**Keywords:** fatty liver, laparoscopic pancreatoduodenectomy, long‐term outcomes, nutrition, sarcopenia

## Abstract

**Aim:**

Pancreatoduodenectomy (PD) is a highly invasive surgical procedure associated with postoperative malnutrition. Laparoscopic pancreatoduodenectomy (LPD) is a minimally invasive alternative, but its long‐term effects on nutritional outcomes remain unclear. This study aimed to compare long‐term nutritional outcomes between LPD and open PD (OPD) and to identify factors influencing postoperative nutritional status.

**Methods:**

A retrospective analysis was conducted on 65 patients who underwent PD. Nutritional indicators, including the psoas muscle index (PMI), prognostic nutritional index, and liver‐to‐spleen ratio, were assessed at 3, 6, and 12 months postoperatively. Multivariate analysis was performed to determine factors affecting nutritional outcomes.

**Results:**

The LPD group (*n* = 36) demonstrated better PMI preservation at 12 months compared with the OPD group (*n* = 29) (*p* = 0.002), with significantly lower fatty liver incidence (3.7% vs. 22.7%, *p* = 0.038) and higher prognostic nutritional index values at 3 months (*p* = 0.029). LPD was identified as an independent factor for improved PMI (*p* = 0.020). Additionally, LPD was associated with reduced blood loss and shorter hospital stays.

**Conclusion:**

LPD improves long‐term nutritional outcomes by preserving muscle mass and reducing metabolic disruptions, thus supporting its role in enhancing postoperative recovery and quality of life. Further prospective studies are warranted to confirm these findings.

## Graphical abstract

Minimally invasive laparoscopic pancreaticoduodenectomy (LPD) significantly improves long‐term nutritional outcomes compared to open pancreaticoduodenectomy (OPD). LPD patients demonstrated better preservation of muscle mass index (PMI) and a lower incidence of postoperative fatty liver. The reduced surgical trauma associated with LPD contributed to enhanced postoperative nutritional status and quicker recovery.

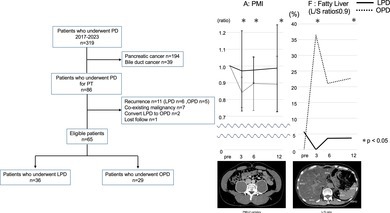

## 1 Introduction

Pancreatoduodenectomy (PD) is a surgical procedure for managing tumors in the pancreatic head, periampullary region, and distal bile duct. This procedure has significant challenges due to its involvement with multiple organs, including the pancreas, duodenum, bile duct, and stomach, as well as the proximity to major blood vessels. Over recent decades, advancements in surgical technology have reduced hospital mortality rates to ≤ 5% [1]. However, PD remains associated with major postoperative complications, even at high‐volume centers. Its invasive nature, coupled with postoperative complications and resultant exocrine pancreatic insufficiency, often contributes to malnutrition [2]. This postoperative malnutrition significantly impairs patients' quality of life (QOL) [3, 4] and may delay their return to normal social and occupational activities.

Laparoscopic pancreatoduodenectomy (LPD), first reported by Gagner and Pomp in 1994 [5], is a pioneering advancement in minimally invasive pancreatic surgery. LPD aims to reduce surgical trauma while adhering to oncological principles [6] and potentially improving postoperative recovery [7]. Several studies have demonstrated the safety and feasibility of LPD in terms of short‐term outcomes, such as reduced blood loss, shorter hospital stays, and lower rates of immediate postoperative complications [8, 9, 10, 11]. However, long‐term nutritional outcomes comparing LPD with open pancreatoduodenectomy (OPD) remain poorly understood. Although both procedures involve similar extents of resection and reconstruction, differences in the surgical approaches could influence postoperative recovery, gastrointestinal function, and ultimately, nutritional status. This study aimed to compare the long‐term nutritional outcomes between patients undergoing LPD and OPD to guide surgical decision‐making and improve postoperative nutritional management, ultimately enhancing patient care and QOL after PD.

Nutritional management after PD is a unique challenge. Reconstruction of the pancreas and bile duct often disrupts the digestion and absorption of nutrients, with fat and fat‐soluble vitamin malabsorption being particularly concerning due to reduced pancreatic enzyme and bile secretion. This increases the risk of malnutrition over time [12]. Additionally, limited food intake resulting from postoperative complications, appetite loss, and early satiety may further delay recovery [13]. Appropriate nutritional management and support are essential. Understanding the impact of different surgical approaches on postoperative nutritional outcomes may enhance nutritional care strategies. This study is the first to evaluate long‐term nutritional differences between LPD and OPD, aiming to improve QOL and clinical outcomes for patients recovering from PD.

## 2 Methods

### 2.1 Study Design and Patient Population

This retrospective observational study used a prospectively maintained institutional database. The ethics committee of Kyoto University Graduate School and Faculty of Medicine (approval code: R1721‐3) approved the study protocol, which adhered to the provisions of the Declaration of Helsinki. Informed consent was obtained from all patients using the opt‐out method. This study included consecutive patients who underwent PD at Kyoto University Hospital between January 2017 and December 2023. To eliminate the impact of lymph node dissection on surgical techniques, patients with pancreatic cancer and bile duct cancer were excluded. Furthermore, to account for the influence of cachexia, patients with postoperative recurrence and those with coexisting malignancies were excluded. Patients who required conversion to open surgery and those who were lost to follow‐up were also excluded. The cohort was divided into two groups based on the surgical approach, either LPD or OPD. In our department, LPD was introduced in 2017 for low‐grade malignant tumor treatment. Following changes in insurance coverage, we expanded the indications for LPD to include cases without vascular resection. The procedures were performed by a supervisor from our department's pancreatic surgery group, either as the primary surgeon or as a supervising assistant. Follow‐up data were updated in May 2024.

### 2.2 Surgical Technique and Postoperative Management

The surgery was performed under general anesthesia using an open abdominal approach. A midline incision in the upper abdomen provided access to the pancreatic head, bile duct, and surrounding structures. Extensive lymphadenectomy around the stomach or aorta was not performed. However, lymphadenectomy was conducted around the hepatic hilum and lymph nodes adjacent to the superior mesenteric artery (SMA). The SMA nerve plexus was carefully preserved unless oncological clearance required resection. The portal and superior mesenteric veins were dissected. Standard resections were followed by reconstruction to restore gastrointestinal continuity, which included pancreatojejunostomy, hepaticojejunostomy, and gastrojejunostomy. In LPD, five ports were inserted in the upper abdomen [14]. Mobilization of the right colon was performed to secure the surgical field. The extent of organ resection and lymphadenectomy matched that of OPD, including resection of the pancreatic head, duodenum, bile duct, and surrounding structures. Lymphadenectomy was similarly conducted around the hepatic hilum and adjacent to the SMA. Reconstruction involved pancreatojejunostomy, hepaticojejunostomy, and gastrojejunostomy (or duodenojejunostomy), depending on intraoperative findings. This was achieved through either a small laparotomy incision or a purely laparoscopic approach. Postoperative management, including the duration of high care unit stay and initiation of oral intake, has been standardized since 2017. After surgery, we started monitoring patients in a high care unit for 2–3 days, with vital signs and urine output assessed every 4–8 h. Early oral intake was initiated after surgery, accompanied by pancrelipase administration at a dose of 1800 mg/day. During the first 3 months after discharge, general condition and blood test results were evaluated every 2–4 weeks. Subsequently, CT imaging and nutritional status were monitored every 3 months.

### 2.3 Data Collection

The clinicopathological characteristics of the patients included age, sex, body mass index (BMI), treatment‐related variables, and survival data. Urine output was measured at 8‐h intervals, with the mean hourly volume of urine adjusted for body weight [15]. Postoperative complications, such as pancreatic fistula and delayed gastric emptying, were recorded based on the definitions provided by the International Study Group of Pancreatic Surgery [16, 17] or the Clavien–Dindo classification [18]. Nutritional indicators included BMI, the prognostic nutritional index (calculated as 10 × albumin [g/dL] + 0.005 × total lymphocyte count [/μL]), the neutrophil‐to‐lymphocyte ratio, and the advanced lung cancer inflammation index (calculated as BMI [kg/m^2^] × albumin [g/dL] × total lymphocyte count [/μL]/total neutrophil count [/μL]) [19]. The psoas muscle index (PMI) was calculated by measuring the cross‐sectional area of the psoas muscles at the level of the L3 vertebra on CT scans and dividing it by the square of the patient's height [20] (Figure S1). The liver‐to‐spleen (L/S) ratio was derived from CT values measured at three points in each organ (Figure S1), with a ratio ≤ 0.9 defined as indicative of fatty liver [21]. Nutritional indicators, PMI, and the L/S ratio were assessed preoperatively and at 3, 6, and 12 months postoperatively. Postoperative nutritional indicators and PMI were expressed as relative values, with preoperative values set to 1.

### 2.4 Primary and Secondary Endpoints

The primary endpoint was the relative PMI value at 12 months after surgery. Secondary endpoints included rates of surgical complications, changes in nutritional indicators, and variations in the L/S ratio.

### 2.5 Statistical Analysis

Continuous variables are expressed as medians, with interquartile ranges to indicate variability. The Kruskal–Wallis test was used to analyze continuous variables, whereas the likelihood ratio test was used to evaluate categorical variables. Variables related to the relative PMI value at 12 months were selected for univariate analysis based on clinical discretion. Those with a *p* < 0.100 were included in a multivariate logistic regression model. Statistical significance was set at *p* < 0.05. All statistical analyses were conducted using JMP 17.0 Pro software (SAS Institute Inc., Cary, NC, United States).

## 3 Results

### 3.1 Patients

**FIGURE 1:**
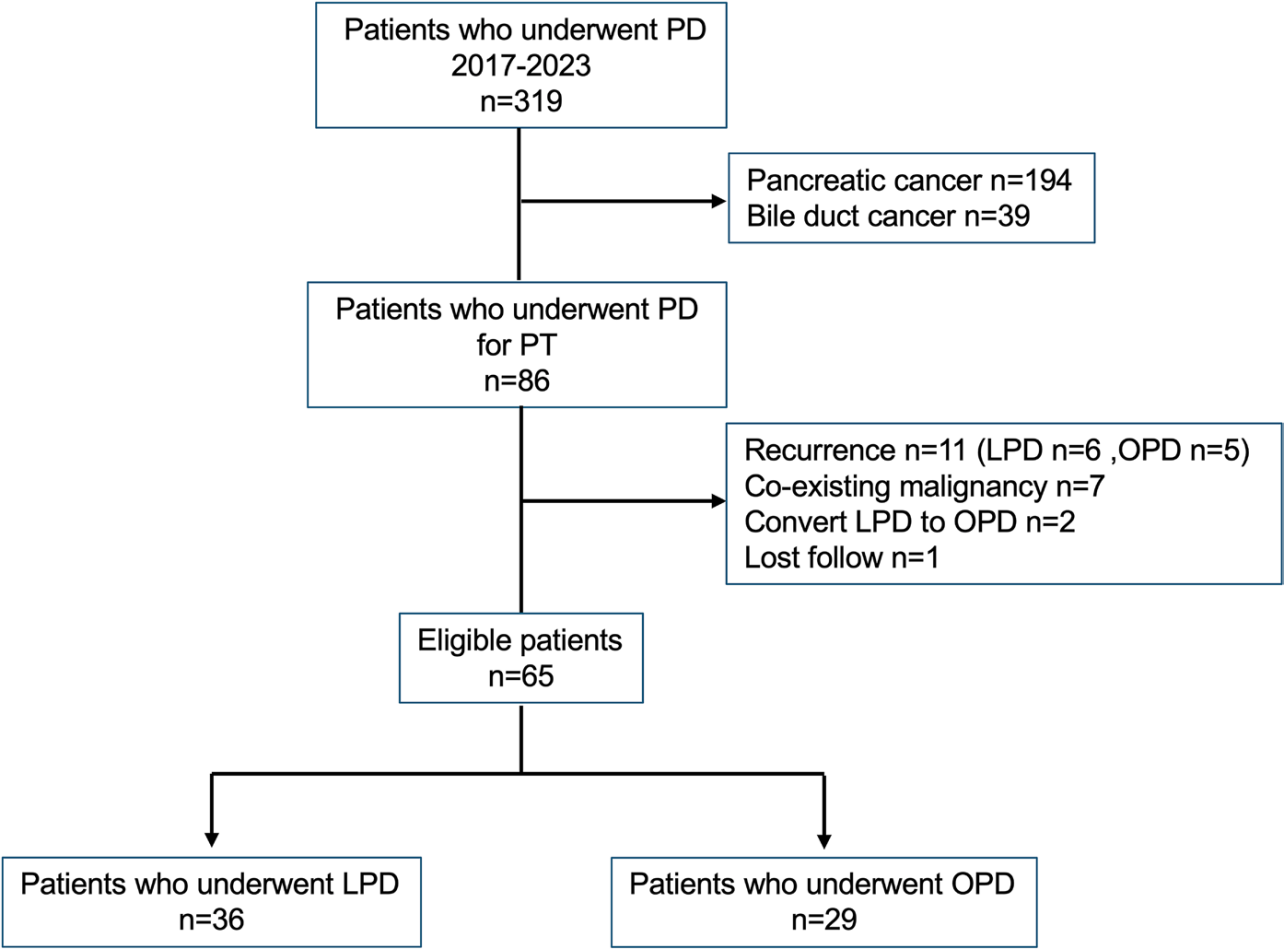
Flow chart of the study. LPD, laparoscopic pancreatoduodenectomy; OPD, open pancreatoduodenectomy; PD, pancreatoduodenectomy; PT, periampullary tumor.

Figure 1 shows a flow chart of the study participants divided into two groups. During the study period, 319 patients underwent PD, with 86 meeting eligibility criteria after excluding cases of pancreatic cancer (*n* = 194) and distal bile duct cancer (*n* = 39). Additional exclusions included patients with recurrence (*n* = 11), coexisting malignancies (*n* = 7), conversion from LPD to OPD (*n* = 2), and those lost to follow‐up (*n* = 1), leaving 65 patients for analysis. Patient demographics are summarized in Table 1. The cohort had a median age of 67 years and a median BMI of 22.5. The most frequent indication for surgery was intraductal papillary mucinous neoplasm (*n* = 23), followed by ampullary carcinoma and neuroendocrine neoplasms. LPD was performed in 36 patients (55.4%), and OPD in 29 patients (44.6%).

**TABLE 1:** Baseline characteristics of all included patients.

Variables	Total (*n* = 65)	LPD (*n* = 36)	OPD (*n* = 29)	*p*
Age, years (IQR)	67 (54–75.5)	71 (52–79)	65 (55–71)	0.345
Sex, male	23 (35.3%)	14 (38.9%)	9 (31.0%)	0.509
BMI, kg/m^2^ (IQR)	22.5 (20.7–24.4)	23.2 (20.8–26.1)	22.3 (20.7–23.7)	0.268
eGFR (IQR)	71.7 (64.1–82.6)	71.6 (64.6–80.8)	71.9 (61.8–84.1)	0.668
LVEF (IQR)	67 (64.2–72.8)	65.5 (62.5–73.2)	67.6 (65.4–73.1)	0.477
Histopathology				
IPMN, *n* (%)	23 (35.4%)	9 (25.0%)	14 (48.3%)	0.507
Ampullary carcinoma, *n* (%)	19 (29.2%)	11 (30.6%)	8 (42.1%)	0.793
Neuroendocrine neoplasms, *n* (%)	14 (21.5%)	9 (25.0%)	5 (17.2%)	0.446
Solid pseudopapillary neoplasm, *n* (%)	4 (6.1%)	3 (8.3%)	1 (3.5%)	0.402
Duodenal cancer, *n* (%)	2 (3.1%)	2 (5.6%)	0 (0%)	0.120
Others, *n* (%)	3 (4.6%)	2 (5.6%)	1 (3.5%)	0.684
Nutritional assessment at diagnosis				
Albumin, g/dL (IQR)	4.2 (4.0–4.4)	4.2 (3.9–4.5)	4.2 (4.0–4.4)	0.979
TLC, /μL (IQR)	1430 (1165–1979)	1462 (1189–1949)	1418 (1045–1986)	0.968
PMI (IQR)	5.7 (4.7–6.3)	5.9 (4.9–6.4)	5.6 (4.6–6.2)	0.578
PNI (IQR)	49.4 (46.3–52.3)	49.0 (45.5–52.1)	49.4 (46.8–52.6)	0.833
NLR (IQR)	2.0 (1.5–2.6)	1.8 (1.4–3.7)	2.0 (1.7–2.5)	0.553
ALI (IQR)	48.8 (34.7–64.5)	53.7 (34.4–73.1)	48.3 (35.3–59.2)	0.518
Fatty liver, *n* (%)	2 (3.2%)	2 (5.6%)	0 (0%)	0.136

Abbreviations: ALI, advanced lung cancer inflammation index; BMI, body mass index; eGFR, estimated Glomerular Filtration Rate; IPMN, intraductal papillary mucinous neoplasm; LPD, laparoscopic pancreatoduodenectomy; LVEF, left ventricular ejection fraction; NLR, neutrophil to lymphocyte ratio; OPD, open pancreatoduodenectomy; PMI, psoas muscle mass index; PNI, prognostic nutritional index; POPF, postoperative pancreatic fistula; TLC, total lymphocyte count.

### 3.2 Demographics of the LPD and OPD Groups

As shown in Table 1, we compared the preoperative characteristics of patients undergoing LPD and OPD, including age, sex, preoperative nutritional parameters, and underlying diseases. No significant differences were observed in age or sex between the two groups. Cases of duodenal cancer were absent in the OPD group. However, no significant differences were found in the distribution of underlying diseases between the groups. Regarding nutritional status, fatty liver cases were observed preoperatively in the LPD group (*n* = 2) but not in the OPD group. Nevertheless, none of the preoperative parameters demonstrated significant differences between the two groups.

### 3.3 Perioperative Outcomes

The perioperative outcomes are shown in Table 2. Although operative time did not differ between the groups, intraoperative blood loss was significantly lower in the LPD group than in the OPD group (85 vs. 470 mL, *p* < 0.0001). Pancreatic texture showed no difference between the two groups; however, the pancreatic duct diameter was significantly smaller in the LPD group than in the OPD group (2 mm vs. 4 mm, *p* = 0.043). Postoperative complications, classified as Clavien–Dindo grade IIIa or higher, were observed in 19.4% of patients in the LPD group and 24.1% of patients in the OPD group (*p* = 0.527). Similarly, postoperative pancreatic fistula occurred in 13.9% of LPD patients and 13.8% of OPD patients (*p* = 0.991). The rates of delayed gastric emptying and thrombosis were higher in the OPD group (2.8% vs. 13.8% and 5.6% vs. 20.7%, respectively), but the differences were not statistically significant (*p* = 0.092 and *p* = 0.062, respectively). The median hospital stay was significantly shorter for patients in the LPD group than in the OPD group (12 days vs. 28 days, *p* < 0.0001).

**TABLE 2:** Perioperative outcomes.

	LPD (*n* = 36)	OPD (*n* = 29)	*p*
Operation time, min (IQR)	496 (461–553)	495 (450–551)	0.746
Intraoperative blood loss, mL (IQR)	85 (50–165)	470 (285–715)	< 0.0001*
Intraoperative infusion volume (IQR)	3500 (2940–4138)	3040 (2605–3750)	0.12
Blood transfusion, *n* (%)	1 (2.8%)	4 (13.8%)	0.098
Soft pancreas, *n* (%)	30 (83.3%)	19 (65.5%)	0.097
Pancreatic duct diameter, mm (IQR)	2 (2–6)	4 (2–8)	0.043*
1POD CRP, mg/dL (IQR)	5.3 (4.1–6.5)	8.6 (6.9–10.7)	< 0.0001*
3POD CRP, mg/dL (IQR)	18.4 (10.9–22.9)	12.4 (6.4–21.0)	0.094
Clavien–Dindo classification ≥ IIIa, *n* (%)	7 (19.4%)	7 (24.1%)	0.527
POPF grade B/C, *n* (%)	5 (13.9%)	4 (13.8%)	0.991
DGE, *n* (%)	1 (2.8%)	4 (13.8%)	0.092
Thrombosis, *n* (%)	2 (5.6%)	6 (20.7%)	0.062
Abdominal abscess, *n* (%)	10 (27.8%)	6 (20.7%)	0.51
Hospital stay, days (IQR)	12 (10–20.5)	28 (20–49.5)	< 0.0001*

Abbreviations: CRP, C‐reactive protein; DGE, delayed gastric emptying; LPD, laparoscopic pancreatoduodenectomy; OPD, open pancreatoduodenectomy; POD, postoperative days; POPF, postoperative pancreatic fistula.

* Significantly different (*p* < 0.05).

**FIGURE 2:**
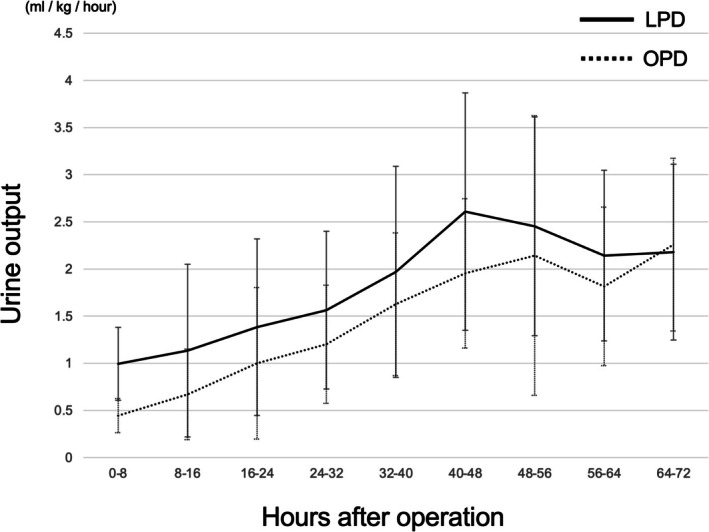
Trends in urine output volume. The average urine volume per body weight was measured every 8 h for 3 days after surgery. Error bars represent the standard error of the mean (SEM). LPD, laparoscopic pancreatoduodenectomy; OPD, open pancreatoduodenectomy.

Urine output within the first three postoperative days consistently remained higher in the LPD group at all time points (Figure 2). In the LPD group, urine output rapidly exceeded 1 mL/kg/h within the first postoperative day and increased to ≥ 2 mL/kg/h within two postoperative days. By contrast, the OPD group required approximately 3 days to achieve urine output exceeding 2 mL/kg/h.

### 3.4 Nutritional Outcomes

**FIGURE 3:**
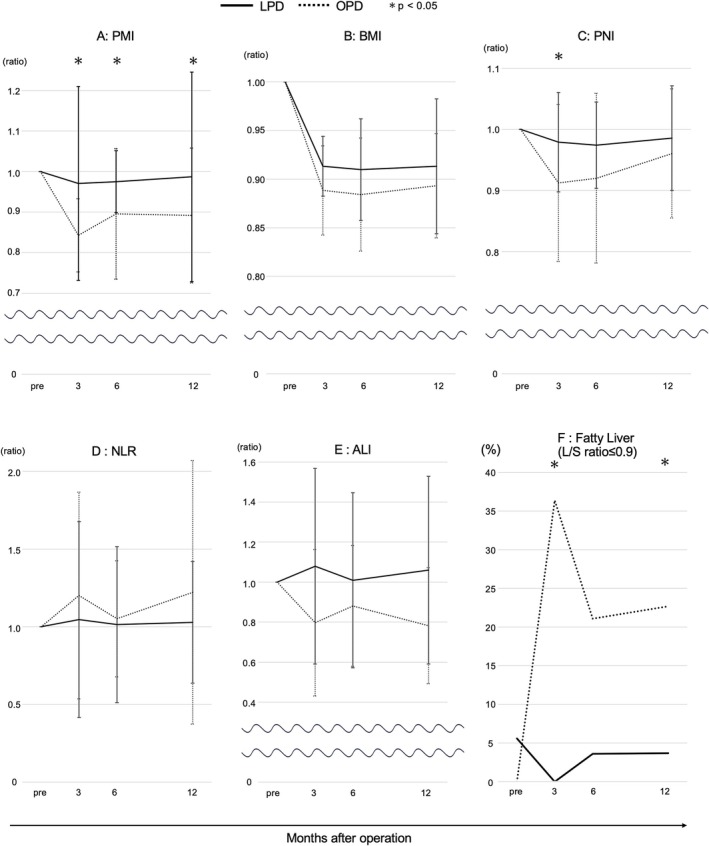
Trends in nutritional scores. (A) PMI, (B) BMI, (C) PNI, (D) NLR, (E) ALI, and (F) Fatty liver. Asterisks indicate data points with significant difference. (A–E) Relative values of the mean scores at 3, 6, and 12 months, with preoperative values normalized to 1. Error bars represent the standard error of the mean (SEM). (F) Progression of fatty liver incidence at each data point over time. ALI, advanced lung cancer inflammation index; BMI, body mass index; L/S ratio, liver‐to‐spleen ratio; LPD, laparoscopic pancreatoduodenectomy; NLR, neutrophil to lymphocyte ratio; OPD, open pancreatoduodenectomy; PMI, psoas muscle mass index; PNI, prognostic nutritional index.

The LPD group showed minimal reduction in PMI rates over time, whereas the OPD group reached a nadir at 3 months postoperatively (0.97 vs. 0.84 and 0.98 vs. 0.90, 0.99 vs. 0.89) and did not return to preoperative levels. Significant differences were observed in PMI rates at 3, 6 months, and 1 year postoperatively (*p* = 0.001, *p* = 0.045, and *p* = 0.002, respectively). BMI rates showed no significant differences between the groups within the first year after surgery. However, the OPD group demonstrated a stronger downward trend (0.91 vs. 0.89 and 0.91 vs. 0.89, 0.90 vs. 0.89). The prognostic nutritional index rate was higher in the LPD group from 3 to 12 months postoperatively (0.98 vs. 0.91 and 0.97 vs. 0.92, 0.98 vs. 0.96), suggesting better postoperative nutritional status, with a significant difference noted at 3 months postoperatively (*p* = 0.029). Although the neutrophil‐to‐lymphocyte ratio and albumin‐lymphocyte index did not show significant differences, the LPD group maintained lower values, reflecting better nutritional status. The incidence of fatty liver, a condition associated with worsened nutritional status, was significantly lower in the LPD group than in the OPD group at 3 and 12 months postoperatively (0% vs. 36.4%, *p* = 0.006 and 3.7% vs. 22.7%, *p* = 0.038, respectively), with the OPD group continuing to show higher rates in subsequent assessments (Table 3). PMI demonstrated the most significant long‐term differences between the LPD and OPD groups (Figure 3).

**TABLE 3:** Trends in nutritional scores.

	PMI (IQR)			BMI (IQR)			PNI (IQR)		
	LPD	OPD	*p*	LPD	OPD	*p*	LPD	OPD	*p*
Pre	5.9 (4.9–6.4)	5.6 (4.6–6.2)	0.578	23.2 (20.8–26.1)	22.3 (20.7–23.7)	0.268	49.0 (45.5–52.1)	49.4 (46.8–52.6)	0.833
3 months	5.5 (4.4–6.1)	4.4 (3.9–5.4)	0.076	21.4 (18.7–23.6)	19.7 (18.4–21.7)	0.214	47.1 (44.9–51.6)	44.6 (39.3–50.2)	0.047*
6 months	5.9 (4.9–6.4)	4.8 (4.1–5.6)	0.347	21.0 (18.7–22.4)	19.8 (18.6–20.8)	0.267	46.9 (44.3–51.4)	46.2 (41.2–50.2)	0.230
12 months	6.0 (4.9–6.4)	5.1 (3.9–5.8)	0.036*	20.8 (19.0–23.1)	20.1 (18.1–21.3)	0.230	48.0 (43.6–50.9)	47.4 (43.1–50.1)	0.477

	NLR (IQR)			ALI (IQR)			Fatty liver (%)		
	LPD	OPD	*p*	LPD	OPD	*p*	LPD	OPD	*p*
Pre	1.8 (1.4–2.7)	2.0 (1.7–2.5)	0.553	53.7 (34.4–73.1)	48.3 (35.3–59.2)	0.518	2/36 (5.6%)	0/26 (0%)	0.136
3 months	1.8 (1.3–2.3)	2.0 (1.7–3.0)	0.072	47.2 (36.6–68.8)	40.0 (22.5–49.2)	0.035*	0/14 (0%)	4/11 (36.4%)	0.006*
6 months	1.8 (1.4–2.2)	2.0 (1.5–2.7)	0.215	49.5 (39.0–62.7)	41.0 (22.8–58.2)	0.167	1/28 (3.6%)	4/19 (21.1%)	0.055
12 months	1.7 (1.4–2.3)	2.2 (1.6–2.6)	0.215	49.7 (32.0–57.8)	33.9 (24.5–53.9)	0.083	1/27 (3.7%)	5/22 (22.7%)	0.038*

Abbreviations: ALI, advanced lung cancer inflammation index; BMI, body mass index; LPD, laparoscopic pancreatoduodenectomy; NLR, neutrophil to lymphocyte ratio; OPD, open pancreatoduodenectomy; PMI, psoas muscle mass index; PNI, prognostic nutritional index.

* Significantly different (*p* < 0.05).

### 3.5 Perioperative Factors for Postoperative Nutritional Status

Perioperative factors affecting postoperative nutritional status were analyzed using logistic regression across all patients (Table 4). The analysis focused on the most significant nutritional indicator: the PMI ratio. The cut‐off value was determined based on the PMI ratio at 12 months postoperatively (0.942). A univariate analysis of average relative PMI values at 12 months postoperatively identified LPD (*p* = 0.001), BMI ≤ 22 (*p* = 0.072), and hospital stays ≤ 14 days (*p* = 0.049) as factors with *p* < 0.01. Multivariate analysis revealed that the surgical approach (LPD vs. OPD) was the only factor with a statistically significant difference, displaying the lowest *p* value (*p* = 0.020). This result suggests that the laparoscopic approach is an independent surgical factor for postoperative nutritional status.

**TABLE 4:** Univariate and multivariate analysis associated with PMI ratio (12 months) ≥ 0.942.

Variable	Univariate analysis		Multivariate analysis	
	OR (95% CI)	*p*	OR (95% CI)	*p*
Laparoscopy	0.156 (0.047–0.515)	0.001*	0.150 (0.030–0.738)	0.020*
Age < 60 years	1.619 (0.516–5.082)	0.406	—	—
BMI ≤ 22 kg/m^2^	0.363 (0.118–1.111)	0.072	0.252 (0.063–2.892)	0.052
Soft pancreas	1.353 (0.399–4.587)	0.628	—	—
Bleeding > 500 mL	0.486 (0.132–1.787)	0.273	—	—
Operation time > 480 min	0.765 (0.318–2.931)	0.950	—	—
Clavien–Dindo classification ≥ IIIa	0.760 (0.192–3.008)	0.696	—	—
Blood transfusion	1.643 (0.140–19.287)	0.687	—	—
Hospital stay > 14 days	0.230 (0.068–0.779)	0.013*	0.521 (0.094–2.892)	0.456

Abbreviations: BMI, body mass index; PMI, psoas muscle index.

* Significantly different (*p* < 0.05).

## 4 Discussion

This study analyzed data from patients undergoing PD at a high‐volume pancreatic surgery center (Kyoto University) in Japan to evaluate the impact of LPD on both short‐ and long‐term outcomes. The findings demonstrated that the minimally invasive nature of LPD contributes significantly to the improvement of long‐term postoperative nutritional status. Maintaining nutritional health following PD is strongly influenced by reduced surgical invasiveness and effective postoperative nutritional management. In this study, we evaluated preoperative and postoperative nutritional indices using relative values, showing a trend toward improved nutritional outcomes in the LPD group compared with the OPD group. These results highlight the critical role of the reduced invasiveness of LPD in supporting long‐term nutritional health after PD.

LPD has demonstrated remarkable benefits in maintaining favorable nutritional indices and preserving muscle mass up to 1 year postoperatively, a feature that stands out as a key advantage. By effectively minimizing postoperative changes in body composition, LPD not only supports better physical function and smoother recovery but also plays a pivotal role in enabling the continuation of adjuvant chemotherapy and promoting early reintegration into daily life. Furthermore, the preservation of muscle mass has the potential to significantly enhance long‐term prognostic outcomes, providing patients with a stronger foundation for recovery and overall well‐being. As secondary sarcopenia caused by surgical stress has been shown to adversely affect physical function and the feasibility of adjuvant therapies, as reported by Kaido et al. and Oh et al. [22, 23], the muscle‐sparing effect of LPD emerges as a transformative approach. These findings underscore the critical role of LPD in both short‐term recovery and long‐term treatment strategies, establishing it as a cornerstone of comprehensive perioperative and extended care, offering patients substantial and enduring benefits.

Postoperative fatty liver following PD can occur through various mechanisms, including nutritional deficiencies, inflammation, and metabolic disruptions. Impaired fat absorption after PD, a result of pancreatic exocrine insufficiency, leads to nutritional deficits that promote hepatic fat accumulation [24]. Surgical trauma triggers systemic inflammation and increases stress hormones, such as cortisol, which promote fat storage in the liver [25]. Altered bile acid metabolism and changes in gut microbiota after PD further disrupt lipid metabolism, increasing the risk of fatty liver [26]. In this study, the LPD group exhibited a significantly lower incidence of postoperative fatty liver compared with the OPD group. This difference may be attributed to the minimally invasive nature of LPD, which reduces surgical trauma and inflammation, thus minimizing stress hormone levels and metabolic disturbances. Faster recovery and improved nutritional status in the LPD group likely contributed to better lipid metabolism, protecting against the development of fatty liver disease.

Consistent with previous reports, the minimally invasive nature of LPD is associated with reduced intraoperative blood loss [27]. Postoperative trends in urine output also indicated better fluid balance and reduced stress on renal function in the LPD group than in the OPD group, suggesting a lower physiological burden. Notably, since there were no significant differences in preoperative renal and cardiac function or intraoperative fluid administration between the two groups, this difference is primarily attributable to the surgical approach. Urine output often decreases during the intraoperative and postoperative periods due to the release of aldosterone and vasopressin, which are triggered by surgical stress, hypovolemia, or anesthesia [28]. This decrease represents a common physiological response to stress during surgery. By mitigating these stress responses, LPD demonstrates potential for reducing perioperative complications, promoting early stabilization of circulatory dynamics, and contributing to improved overall clinical outcomes.

The advantages of minimally invasive surgery (MIS) on postoperative nutritional status have been demonstrated in various organs. For example, in colorectal resection, MIS facilitates quicker recovery of gastrointestinal function, reduces postoperative pain, and allows for earlier resumption of oral intake, which ultimately supports better nutritional outcomes [29]. Similarly, laparoscopic gastric surgeries, such as partial or total gastrostomies, improve postoperative nutritional status and muscle mass volume compared with open surgery. These benefits are likely attributed to reduced surgical trauma and faster recovery times [30]. These findings highlight the positive impact of laparoscopic procedures not only on surgical recovery but also on nutritional outcomes across various organ systems, making MIS a key consideration for improving overall patient care.

This study had several limitations. First, the retrospective nature of the study and the relatively small sample size limit the generalizability of the findings. Second, the lack of standardized evaluation criteria for postoperative nutritional indices and sarcopenia presents methodological challenges. Third, surgeon‐related factors represent another limitation of this study. All LPD procedures were performed either by an experienced pancreatic surgeon from our department as the primary surgeon or as a supervising assistant, which may influence the generalizability of the findings. In addition, the increasing adoption of robot‐assisted surgery as a modern surgical approach highlights the need for future studies to evaluate the impact of minimally invasive approaches, including robot‐assisted PD, on postoperative nutritional status and sarcopenia. Large‐scale prospective studies are warranted to address these limitations and to establish uniform criteria for assessing outcomes related to nutrition and sarcopenia across different surgical modalities.

In conclusion, LPD is a promising surgical approach that combines the advantages of minimal invasiveness with improved short‐term postoperative outcomes and long‐term maintenance of nutritional and physical health. The results of this study suggest that LPD may enhance both the QOL and survival outcomes for patients undergoing PD. Further investigations are essential to validate these findings and explore the advantages of this technique.

## Author Contributions

**Koki Kurahashi:** conceptualization, data curation, formal analysis, investigation, methodology, project administration, resources, software, validation, visualization, writing – original draft, writing – review and editing. **Takayuki Anazawa:** conceptualization, data curation, formal analysis, investigation, methodology, project administration, resources, software, supervision, validation, visualization, writing – original draft, writing – review and editing. **Kei Yamane:** conceptualization, data curation, formal analysis, funding acquisition, investigation, methodology, project administration, resources, software, supervision, validation, visualization, writing – original draft, writing – review and editing. **Kazuyuki Nagai:** conceptualization, formal analysis, investigation, methodology, project administration, resources, software, validation, visualization, writing – review and editing. **Satoshi Ishida:** conceptualization, data curation, formal analysis, investigation, methodology, project administration, resources, software, validation, visualization, writing – review and editing. **Satoshi Ogiso:** conceptualization, formal analysis, investigation, methodology, project administration, resources, supervision, validation, visualization, writing – review and editing. **Yoichiro Uchida:** conceptualization, formal analysis, investigation, methodology, resources, validation, visualization, writing – review and editing. **Takashi Ito:** conceptualization, formal analysis, investigation, methodology, project administration, resources, supervision, validation, visualization, writing – review and editing. **Takamichi Ishii:** conceptualization, formal analysis, funding acquisition, investigation, methodology, project administration, resources, validation, visualization, writing – review and editing. **Etsuro Hatano:** conceptualization, formal analysis, investigation, methodology, project administration, resources, supervision, validation, visualization, writing – review and editing.

## Ethics Statement

The study was approved by the Ethics Committee of the Graduate School and Faculty of Medicine, Kyoto University (approval no.: R1721‐3) and was carried out in compliance with the Helsinki Declaration.

## Consent

Informed consent was obtained from all the patients using the opt‐out method.

## Conflicts of Interest

The authors declare no conflicts of interest.

## Supporting information


**Figure S1.** Cross‐sectional computed tomographic images estimating (A) liver‐to‐spleen (L/S) ratio (B) psoas muscle index (PMI) at the L3 vertebral level.
