# Supplementary material for: Long‐Term Nutritional Benefits of Laparoscopic Pancreatoduodenectomy Over Open Surgery
Source: Ann Gastroenterol Surg. 2025 May 26;9(6):1263–72. doi: 10.1002/ags3.70038 (PMC12586923; doi:10.1002/ags3.70038)
Supplement: Supplementary file 1 — Figure S1. Cross‐sectional computed tomographic images estimating (A) liver‐to‐spleen (L/S) ratio (B) psoas muscle index (PMI) at the L3 vertebral level. [file AGS3-9-1263-s001.docx]

**Supporting Information**

Supplemental Figure S1. Cross-sectional computed tomographic images A:L/S ratio (average CT values calculated from three measurement points at the liver and spleen). B: PMI (cross-sectional CT images at the L3 vertebral level, with the bilateral psoas muscle areas measured through manual tracing)
